# Atraumatic Dislocation After Arthroscopic Capsular Release in a Patient With Pseudocholinesterase Deficiency

**DOI:** 10.5435/JAAOSGlobal-D-24-00135

**Published:** 2025-04-15

**Authors:** John Tyler, Clare K. Green, John P. Scanaliato, Michael Gordon, Steven Kieb, Nata Parnes

**Affiliations:** From the Department of Orthopedics, Carthage Area Hospital, Carthage, NY (Mr. Tyler, Dr. Gordon, Mr. Kieb, and Dr. Parnes); the Department of Orthopedics, Claxton-Hepburn Medical Center, Ogdensburg, NY (Mr. Tyler, Mr. Kieb, and Dr. Parnes); the George Washington University School of Medicine, Washington, DC (Ms. Green); and the Midwest Orthopaedics at Rush University Medical Center, Chicago, IL (Dr. Scanaliato).

## Abstract

A 68-year-old woman underwent arthroscopic capsular release and manipulation under general anesthesia for adhesive capsulitis. Six hours postoperatively, she suffered an anterior glenohumeral joint dislocation with passive forward flexion while working with physical therapy. Postoperative workup revealed pseudocholinesterase deficiency. Pseudocholinesterase deficiency may increase the risk of atraumatic glenohumeral dislocation in the immediate postoperative period. Caution should be taken concerning early physical therapy in these patients to minimize the risk of dislocation.

Glenohumeral joint (GHJ) dislocation following arthroscopic capsular release (ACR) or manipulation under anesthesia (MUA) is exceedingly rare, with few cases reported in the literature. Gobezie et al^1^ described a traumatic dislocation resulting from a fall following ACR. Similarly, a 1982 study by Haines and Hargadon reported two postoperative dislocations in a series of 612 closed MUAs.^2^ To our knowledge, no studies have described atraumatic glenohumeral dislocation following ACR and MUA in the setting of pseudocholinesterase deficiency (PCD).

PCD is defined by low plasma cholinesterase levels resulting in impaired metabolism of choline esters. Patients with PCD experience prolonged paralysis following systemic neuromuscular blockade with depolarizing muscle relaxants, such as succinylcholine.^3^ Secondary to inhibited biofeedback, PCD may pose challenges when initiating early physical therapy (PT) following a surgical procedure. Regarding the GHJ, aberrant muscular activation has been linked to numerous pathologies, including rotator cuff (RC) tears and instability.^4^ We present a 68-year-old female patient with PCD who suffered atraumatic glenohumeral dislocation six hours postoperatively following ACR and MUA during passive range of motion (ROM) with PT.

The patient was informed that data concerning the case would be submitted for publication and provided consent.

## Case Report

A 68-year-old woman presented to our clinic for left shoulder pain and joint stiffness consistent with adhesive capsulitis. On examination, passive and active ROM was limited to 90° of forward flexion (FF), 0° of external rotation (ER), and internal rotation (IR) to L4. End ROM was tethered in nature, and the patient reported 8 of 10 pain at the terminal 20° in each plane of motion. Sensory and motor function examinations were normal. MRI revealed edema and thickening of the inferior glenohumeral ligament with no evidence of glenohumeral arthritis. Her symptoms persisted following 18 months of conservative management, including PT, nonsteroidal anti-inflammatory medications, and an intra-articular steroid injection, at which point, the decision was made to proceed with left shoulder ACR, arthroscopic subacromial decompression, and MUA.

General anesthesia was used for the case, and per review of the anesthesia records, 100 mg of succinylcholine was administered intravenously during induction to achieve paralysis. The patient was then positioned in a modified beach chair position. Evaluation under anesthesia demonstrated shoulder motion in FF of 90°, ER of 0°, and IR of 40°. Diagnostic arthroscopy revealed a markedly thickened capsule both anteriorly and posteriorly, as well as a markedly scarred and contracted rotator interval. In addition, a type II superior labrum anterior-posterior lesion was noted, as well as marked inflammation of the biceps tendon and severe subacromial bursitis. No other abnormalities of the GHJ or subacromial space were observed. Following diagnostic arthroscopy, the long head of the biceps tendon was transected, and the rotator interval was released, followed by the release of the anterior capsule from the rotator interval down to the 5-o'clock position. The posterior capsule was then released from just posterior to the biceps tendon down to the 7-o'clock position. Attention was then turned to the subacromial space, and a thorough subacromial bursectomy was performed. The shoulder was gently manipulated, and open, subpectoral biceps tenodesis was completed as the final step of the case. Postoperatively, ROM was noted to be 160° of FF, 100° of abduction, and 70° of ER. With the arm abducted 90°, there was 90° of ER and 60° of IR. No intraoperative complications were found; however, following discontinuation of anesthesia, the patient required respiratory support for 90 minutes before extubation. The patient was transferred to the postoperative care unit in stable condition.

In our practice, PT evaluates patients and begins passive ROM exercises in the postoperative care unit following ACR and MUA. Six hours postoperatively, the patient was noted by PT to have full muscle power in FF, abduction, ER, and IR in static examination. However, on first attempt to initiate passive FF with PT, the patient's shoulder dislocated anteriorly at 150° of FF.

Radiograph confirmed anterior-inferior dislocation of the left GHJ. Propofol was administered for sedation, and the shoulder was reduced without complication. The patient was neurovascularly intact on postreduction examination and subsequently discharged to home with a shoulder immobilizer. Twenty-four hours later, reevaluation revealed a stable shoulder with full passive ROM. The patient then started rehabilitation protocol, focusing on passive and active ROM exercises. Gradual strengthening began at 6 weeks postoperatively.

At 3 months follow-up, she had full active and passive ROM with FF to 160°, ER to 70°, and IR to T7. Full strength and normal sensation were noted on examination. Shoulder apprehension and relocation tests were negative. Two weeks postoperatively, a diagnosis of PCD was confirmed through genetic testing. Favorable clinical outcomes were well maintained at 2 years postoperatively.

## Discussion

PCD is a genetic or acquired defect in the pseudocholinesterase enzyme with an estimated homozygote incidence of 1 in 2,000 to 1 in 5,000 and heterozygote incidence of 1 in 500. The inherited form stems from a mutation in the butyrylcholinesterase gene. Acquired PCD may be secondary to conditions, including hepatic impairment, renal disease, malignancy, and certain medications.^5-7^ Pseudocholinesterase is required to metabolize succinylcholine,^8^ and the degree and longevity of neuromuscular blockade depend on endogenous concentration and functional activity.^8^ The duration of action following standard dosing is typically 5 to 10 minutes. However, heterozygotes for the PCD mutation experience an approximately 30% increase in duration of blockade, and homozygotes have an increased duration of 2 to 3 hours.^9,10^

Currently, there is no cure for the genetic variants of PCD. Patients with PCD who receive succinylcholine require prolonged ventilatory support until choline esters are metabolized, and neuromuscular function is regained.^9,11^ However, the use of succinylcholine in patients with PCD may present additional challenges in patients undergoing shoulder surgery. Despite full recovery of respiratory function and seemingly normal RC muscle power on static examination, we suspect that our patient's neuromuscular control and biofeedback were still partially inhibited at the time of evaluation by PT, resulting in atraumatic glenohumeral dislocation. A few hours following reduction, the shoulder was stable through active and passive ROM, and the patient did not experience any subsequent instability throughout her recovery.

GHJ stability is provided by capsular and ligamentous structures, articulating surfaces, and the synchronous activation of shoulder musculature. Each of these structures is essential to joint stability, as no single structure stabilizes the shoulder in all positions.^12^ The synchronous activity of shoulder musculature, primarily the RC, functions to restrain the humeral head and facilitate normal kinematics, allowing for joint stability. The role of shoulder musculature is particularly evident during the middle ranges of motion, when the capsuloligamentous structures are lax, leaving the RC muscles as the primary force acting to center the humeral head in the glenoid fossa.^13^ Notably, our patient dislocated at slightly past midrange FF, further supporting our hypothesis that residual blockade impairing the RC muscles likely contributed to instability during passive FF.

Although we believe that this patient's isolated instability event was most likely the result of inhibited neuromuscular control and biofeedback secondary to underlying PCD, we recognize that iatrogenic instability cannot be ruled out completely. ACR may impair neuromuscular control by destroying capsule mechanoreceptors,^14-16^ potentially compromising shoulder proprioception and resulting in reduced joint stability.^17^ However, reports of glenohumeral dislocation following ACR or MUA are exceedingly rare in the literature,^1,2^ suggesting that resulting deficits in proprioception do not commonly result in symptomatic instability. Concerning the concomitant biceps tenodesis, biomechanical studies have suggested that the biceps tendon may act as a secondary stabilizer for the GHJ.^18^ Nevertheless, favorable outcomes are reported following combined biceps tenodesis and anterior labral repair in patients with recurrent instability, suggesting that the absence of an intact biceps insertion does not lead to clinically significant destabilization of the GHJ.^19^ In the present case, our patient did not experience recurrent instability throughout her recovery, and the shoulder was stable to provocative testing on physical examination. She reported excellent outcomes at the 2-year follow-up. Altogether, we believe that the isolated nature of this patient's instability in the immediate postoperative period supports impaired biofeedback and neuromuscular control secondary to prolonged neuromuscular blockade as the most likely etiology.

Although there is a paucity of the literature concerning PCD-associated complications following other orthopaedic procedures, this case has relevance beyond the subspecialty of shoulder and elbow. Regarding total joints, early ambulation has become a commonly used modality following total hip and knee arthroplasty.^20^ However, it may be appropriate to delay PT in patients with suspected PCD to minimize the risk of adverse events secondary to impaired neuromuscular control and biofeedback, similar to those observed in the present case.

In conclusion, PCD may be associated with a risk of atraumatic dislocation following ACR and MUA. In patients with suspected PCD who have received succinylcholine, it may be advantageous to delay early passive ROM. Screening for PCD before general anesthesia could help identify affected individuals, allowing for the avoidance of choline esters or the postponement of passive ROM as needed.
